# No relationship between baseline salivary alpha-amylase and State-Trait Anxiety Inventory Score in drug-naïve patients with short-illness-duration first episode major depressive disorder: An exploratory study

**DOI:** 10.4317/jced.53631

**Published:** 2017-04-01

**Authors:** Joanna Szarmach, Wiesław-Jerzy Cubała, Jerzy Landowski, Anna Chrzanowska

**Affiliations:** 1 Department of Psychiatry, Medical University of Gdańsk, Gdańsk, Poland; 2 Radiometer Sp. z o.o., Warsaw, Poland

## Abstract

**Background:**

Salivary α-amylase (sAA) activity alternations are observed in major depressive disorder (MDD) being associated with depression severity and its specific psychopathological dimensions with anxiety being attributed to distress. No data is available on sAA in MDD according to Hamilton Rating Scale for Depression (HAMD-17) and State-Trait Anxiety Inventory (STAI). The exploratory study examines whether and to what extent baseline sAA level is interrelated to the psychopathological features including severity of symptoms and specific psychopathological dimensions.

**Material and Methods:**

The basal, non-stimulated sAA activity was studied in 20 non-late-life adult, treatment-naïve MDD patients with short-illness-duration and in 20 age- and sex-matched healthy controls along with psychometric assessments with Hamilton Rating Scale for Depression (HAMD-17) and Spielberger State-Trait Anxiety Inventory (STAI).

**Results:**

Significantly lower (*p*=0.011) sAA activity was observed in MDD as compared to controls. No significant correlations were observed between sAA activity and the total HAMD-17 score as well as with regard to the specific core depression, insomnia, anxiety and somatic HAM-D psychopathological dimensions. No significant correlations were also found between sAA and STAIX-1 and STAIX-2 scores.

**Conclusions:**

Low baseline sAA levels in MDD with no correlations between sAA and psychopathological features including severity of symptoms and specific psychopathological dimensions was found.

** Key words:**Salivary alpha-amylase, major depressive disorder, Spielberger State-Trait Anxiety Inventory, Hamilton Rating Scale for Depression.

## Introduction

Major depressive disorder (MDD) is often associated with an altered monoamine neurotransmission accompanied by hypothalamic-pituitary-adrenal (HPA) axis dysfunction and autonomic nervous system (ANS) maladaptive activation, being all associated with chronic stress. Salivary α-amylase (sAA) is adopted as a marker of autonomic activation and reflects central noradrenergic activity (1). It is sensitive to physiological stressors. Baseline sAA levels are significantly associated with chronic stress and stress reactivity in healthy individuals (1) with marked increases in sAA due to acute psychological stress and subjective anxiety reports. Changes in sAA were also seen in psychopathology, especially in anxiety-related disorders (2).

It is hypothesised that the elevated sAA is present in MDD being indicative of an increased ANS activity. There is, however, considerable inconsistency in the results attributing sAA elevation to illness stage, severity, specific dimensions of depression and measures of distress (3,4). The elevated sAA activity was associated with depression severity and specific psychopathological dimensions of MDD with anxiety being attributed to distress. STAI performance has been used as an indicator of general anxiety, general psychological distress and general emotional distress. Nonetheless, systematic clinical data on sAA levels in MDD according to STAI profile are limited to a single study in 71 depressed patients where sAA levels were significantly increased in unremitted patients with MDD compared to both healthy controls and remitted patients with MDD. Still, STAI scores (State or Trait) were not associated with sAA level although STAI measurements in unremitted patients with MDD were significantly increased compared with healthy controls and remitted patients (3). Thus anxiety levels in MDD might not be associated with sAA or salivary cortisol levels.

Recently we demonstrated low baseline sAA in drug-naïve patients with short-illness-duration first episode MDD where sAA activity was not significantly correlated neither with duration nor the severity of depressive symptoms as measured by the total HAMD-17 score (5). This exploratory study was undertaken to examine whether and to what extent sAA activity is interrelated to the psychopathological features of MDD including severity of symptoms and specific psychopathological dimensions in baseline non-stimulated conditions. It was hypothesized that the sAA elevation would be correlated with the measures of severity and distress in MDD in non-challenging and stress-free setting.

## Material and Methods

-Subjects

The study population has been described in detail elsewhere (5). Briefly, 20, first-episode drug-naïve MDD patients were recruited and diagnosed with the Structured Clinical Interview for DSM-IV Axis I Disorders (6). The depression severity was rated with 17-item Hamilton Rating Scale for Depression (HAMD-17) (7). Subjects with HAMD-17 score of ≥20 and episode duration ≤24 weeks were eligible. Anxiety was assessed with the Spielberger State-Trait Anxiety Inventory (STAI) (8). Exclusion criteria were: any other Axis I disorder, psychotic symptoms, suicidality, somatic comorbidity, history of oral health problems including salivary gland disorders, concomitant medication including dietary supplements, hormonal contraception, pregnancy/lactation, BMI ≤18 and ≥30, age <18 and >55 years.

The control group consisted of 20 healthy subjects matched by age, sex, and metabolic parameters being naïve to psychotropic drugs. They were interviewed with the Structured Clinical Interview for DSM-IV, nonpatient edition (6). All subjects underwent routine physical examination and were administered STAI inventory (8). A HAMD-17 score ≤5 was required for inclusion. None of them had a history of serious somatic disease or a family history of major psychiatric illness in their first-degree relatives.

The study was performed in agreement with the Declaration of Helsinki following the approval of the Ethic Research Committee of the Institution. For each study participant, written consent was obtained.

-Procedure and measures

The study followed a case-control design. As previously described (5) saliva was sampled and processed with subsequent batch sAA activity analysis by means of an enzyme-linked immunoassay using an ELISA kit (Salivary α-Amylase ELISA Kit, Salimetrics LLC, USA).

The STAI results were analysed with regard to the differentiation of the anxiety to anxiety caused by a specific condition (state subscale - STAIX-1), and anxiety as a more permanent characteristics of the personality (trait subscale - STAIX-2) (8). The total HAMD-17 score was analysed followed by the exploratory analysis based on the hierarchical Cole and Motivala model (9) with core depression, insomnia, anxiety and somatic psychopathological dimensions.

-Statistical analysis

Statistical procedures were performed using StatsDirect v2.7.9. Shapiro-Wilk test was used to assess normal distribution of continuous data. Normally distributed variables were compared using Student’s t-test, all other continuous data were compared with nonparametric Mann-Whitney U-test. The Spearman rank correlation coefficient was used to assess correlations between the obtained variables. All tests were two-tailed with an alpha=0.05.

## Results

The sAA was significantly lower in MDD as compared to controls (*p*=0.011). Significantly higher STAIX-1 (*p*<0.0001) and STAIX-2 (*p*<0.0001) scores were observed in MDD as related to controls. There were no significant differences in terms of gender, age, BMI or WHR between MDD patients and controls with none of these factors being significantly associated with sAA. Additional information can be found in prior reports (5,10).

The sAA in MDD subjects was not significantly correlated neither with duration nor severity of depressive symptoms as measured by the total HAMD-17 score nor analysed psychopathological dimensions. No significant correlations were found between sAA activity and STAIX-1 and STAIX-2 scores ( Table 1).

**Table 1:**
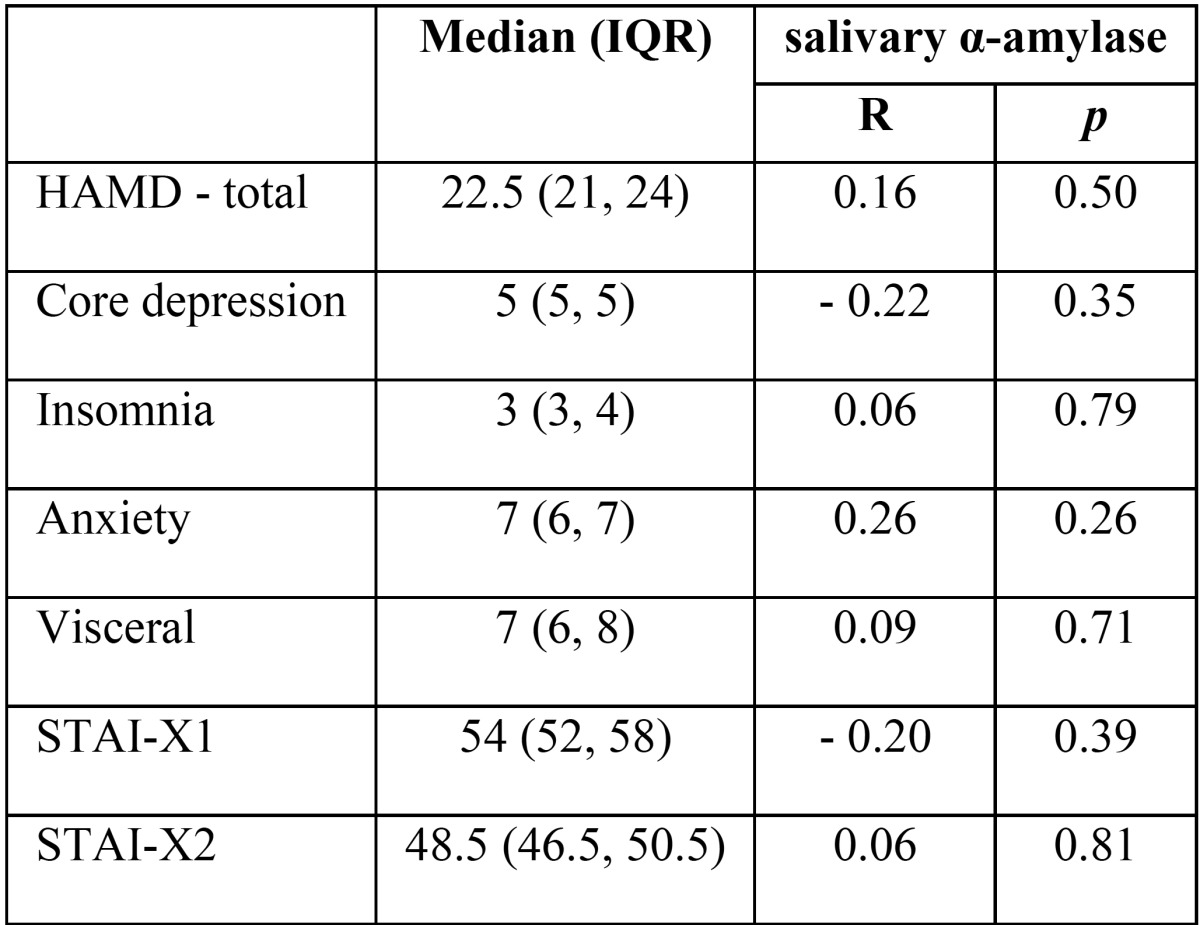
Pearson’s correlation coefficient between sAA activity and psychometric variables in MDD.

## Discussion

Low baseline sAA was seen in depressed individuals as compared to controls. The depressive episode duration and global severity were not found to be significantly correlated with sAA activity as previously demonstrated (5). The results do not support the study hypothesis on the link between MDD and sAA elevation being correlated with the measures of severity and distress in MDD.

The relationship between sAA level and stress is complex. Salivary alpha-amylase is highly sensitive to physiological stressors (2) and also psychosocial stress has been shown to lead to a prompt increase in sAA activity (11) with low durability of its stress induced increase rapidly recovering to normal sAA levels after stress reduction in about 10 minutes. Interestingly, the momentary sAA levels are associated with high arousal affective states, regardless of emotional valence. Elevated momentary levels of sAA have also been associated with greater chronic stress (1,12).

The STAI consist two separate, self-report scales for measuring the distinct concepts of state and trait anxiety. It is used as an indicator of general anxiety, general psychological distress and general emotional distress. State anxiety can measure the temporary and situational anxiety state accompanied by autonomic excitement. The Trait scale assesses the tendency of an individual to respond to stressful circumstances under conditions of increased anxiety (8). To date, there are no investigations that incorporated the use of baseline sAA levels and STAI to determine the relationship among distress, anxiety, and clinical characteristics in MDD.

In healthy subjects sAA activity is associated with subjective anxiety reports (2). In self-report anxiety measure with STAI a high correlation between state anxiety measures and sAA release were found when subjects were exposed to aversive stimuli (2,13). Contrarily, a positive correlation among sAA activity, trait anxiety and subjective stress was also observed indicating the association between trait anxiety and sAA level (14).

Changes in sAA were demonstrated in psychopathology, particularly in anxiety-related disorders (2). Scarce data on baseline sAA as related to depressive psychopathology exist. In a study by Wingenfeld *et al.* (15) neither depression nor trait anxiety or percei-ved stress did influence sAA activity in questionnaire data. The sole systematic study on sAA levels in MDD according to STAI profile in a population of 71 depressed patients revealed significantly increased sAA levels in unremitted patients with MDD compared to both healthy controls and remitted MDD patients. Still, STAI (State or Trait) and HAMD-17 scores were not correlated with sAA level although STAI measurements in unremitted patients with MDD were significantly increased compared with healthy controls and remitted patients (3).

The results corroborate with the paper by Ishitobi *et al.* (3) indicating specificity of MDD where anxiety levels in depressed states might not be associated with sAA level. It may be hypothesized that STAI measures are not associated with basal, non-stimulated sAA activity as its secretion occurs in response to acute stress. These results imply that the level of sAA is associated with MDD as the condition rather than distress (5).

Certain study limitations exist as the explorative study design with the observed associations does not represent causal relationships between the investigated parameters and may considerably contribute to the observed results that apply to drug-naïve patients with short-illness-duration first episode MDD who were free of comorbid Axis I and II conditions, current suicidality and suicide history.

## Conclusions

In conclusion, in basal, non-stimulated conditions low morning baseline sAA levels were found in MDD with no correlations observed between sAA and core depression, insomnia, anxiety and somatic psychopathological dimensions nor STAIX-1 and STAIX-2 scores.

A cross-sectional analysis adds to the evidence linking sAA activity with psychopathology of MDD at the early stage of the disease, with no correlations between sAA and psychopathological features, including severity of symptoms and specific psychopathological dimensions.
